# Sciatic neuropathy: findings on magnetic resonance neurography

**DOI:** 10.1590/0100-3984.2015.0205

**Published:** 2017

**Authors:** Paulo Moraes Agnollitto, Marcio Wen King Chu, Marcelo Novelino Simão, Marcello Henrique Nogueira-Barbosa

**Affiliations:** 1 MD, Attending Physician in the Division of Radiology of the Department of Clinical Medicine at Ribeirão Preto Medical School, University of São Paulo (USP), Ribeirão Preto, SP, Brazil.; 2 MD, Radiologist at the Clínica Radiológica WK Diagnose, Taubaté, SP, Brazil.; 3 PhD, Attending Physician in the Division of Radiology of the Department of Clinical Medicine at Ribeirão Preto Medical School, University of São Paulo (USP), Ribeirão Preto, SP, Brazil.; 4 Tenured Associate Professor in the Division of Radiology of the Department of Clinical Medicine at Ribeirão Preto Medical School, University of São Paulo (USP), Ribeirão Preto, SP, Brazil.

**Keywords:** Sciatic nerve, Magnetic resonance imaging, Neuroimaging/methods, Nervous system diseases/diagnosis, Sciatic neuropathy/diagnosis

## Abstract

Injuries of the sciatic nerve are common causes of pain and limitation in the lower limbs. Due to its particular anatomy and its long course, the sciatic nerve is often involved in diseases of the pelvis or leg. In recent years, magnetic resonance neurography has become established as an important tool for the study of peripheral nerves and can be widely applied to the study of the sciatic nerve. Therefore, detailed knowledge of its anatomy and of the most prevalent diseases affecting it is essential to maximizing the accuracy of diagnostic imaging.

## Abstract (pt)

Lesões do nervo ciático são causas comuns de dor e de limitação no membro inferior. Em razão da sua anatomia particular e do seu longo trajeto, o nervo ciático é frequentemente envolvido em doenças da pelve e do membro inferior. Nos últimos anos, a neurografia por ressonância magnética tem se firmado como ferramenta importante para o estudo de nervos periféricos, podendo ser amplamente empregada para o estudo do nervo ciático. Para este fim, o conhecimento detalhado de sua anatomia e das principais doenças envolvidas é fundamental para maximizar o desempenho diagnóstico por imagem.

## INTRODUCTION

Sciatic nerve injuries are common causes of pain and limitation in the lower limbs. Detailed knowledge of the anatomy of the sciatic nerve is essential for the recognition of alterations and diseases involving the nerve^(1,2)^.

In recent years, magnetic resonance imaging (MRI) has established itself as an important tool for the study of peripheral nerves, especially after the development of protocols including sequences optimized for this purpose, generally referred to as magnetic resonance neurography (MRN). Ideally, MRN involves the use of 3.0 T magnets and the protocol includes the following: graded fluid signal-sensitive sequences with fat suppression; a TE > 66 ms; thin slices; and acquisition in the best anatomical planes for study of the nerve or plexus. The use of a TE > 66 ms serves to avoid the magic angle phenomenon, which is related to anisotropy of the peripheral nerve and can simulate signal abnormality. These sequences provide greater spatial and contrast resolution for neural study. MRN reveals peripheral nerve abnormalities by identifying changes in signal intensity, notably on T2-weighted images, together with changes in the cross-sectional area and course of the nerve, as well as disorganization or absence of the typical fascicular pattern. Another focus of MRN-based peripheral nerve studies is the denervation of muscles, an abnormality that can manifest as a pattern of edema or, in chronic cases, hypotrophy of the muscles innervated by the sciatic nerve^(1-4)^.

Clinically, lesions or diseases of the sciatic nerve manifest as pain of varying intensity in the lower lumbar region, with irradiation to the gluteal region and to the posterior region of the ipsilateral lower limb. Those manifestations can be accompanied by changes in sensitivity or motor deficits.

Another MRI technique that can be used for the study of neural pathways is tractography, which involves diffusion-weighted acquisition-typically diffusion tensor imaging (DTI)-and is classically used in studies of the central nervous system^(5-7)^. Recent studies have demonstrated that this technique can also be used in the study of peripheral nerves^(8)^, although we found no studies with an emphasis on the specific evaluation of sciatic nerve abnormalities in humans. Recent experimental studies involving animal models of crush injury and sciatic nerve traction injury showed that DTI tractography, using routine clinical 1.5 T MRI scanners, is a promising tool in the assessment of sciatic lesions^(9,10)^. In those studies, DTI was able to differentiate between nerves with nerve damage and control group nerves. In addition, the values and time curves of fractional anisotropy and the eigenvalue lambda (perpendicular) correlated well with the histological findings of Wallerian degeneration and with functional recovery.

The aim of this essay is to illustrate the imaging aspects of neuropathies and the anatomy of the proximal segment of the sciatic nerve by MRN. Among the causes of neuropathies, we illustrate those that are neoplastic, compressive, traumatic, hereditary, iatrogenic, or idiopathic in nature.

## NORMAL ANATOMY OF THE PROXIMAL SCIATIC NERVE

**Figure 1:**
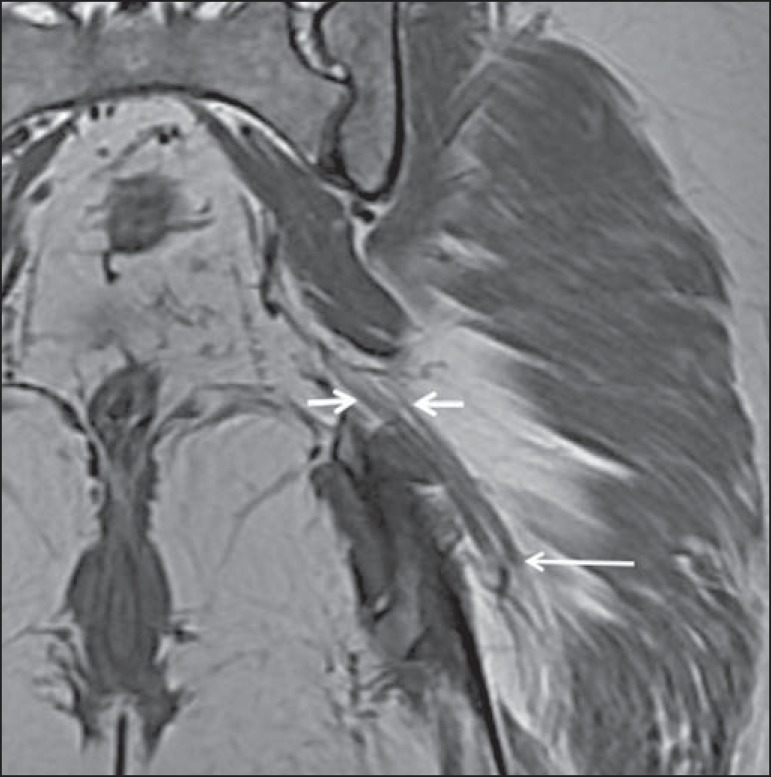
Coronal T1-weighted MRI scan showing the fascicular pattern (long arrow) and perineural fat (short arrows) around the proximal segment of the sciatic nerve.

The lumbosacral plexus is composed of ventral rami of the L4-S3 nerve roots, which join to form the tibial (medial) nerve, the common (lateral) peroneal nerve, and the posterior cutaneous nerve of the thigh. The sciatic nerve consists of the tibial and common peroneal components, which are encased in a common sheath and exit the pelvis through the sciatic notch^(3,4)^, as depicted in Figure 1. Immediately before leaving the pelvis, the sciatic nerve has an intimate relationship with the ventral surface of the piriformis muscle.

## NEOPLASTIC CAUSES

**Figure 2:**
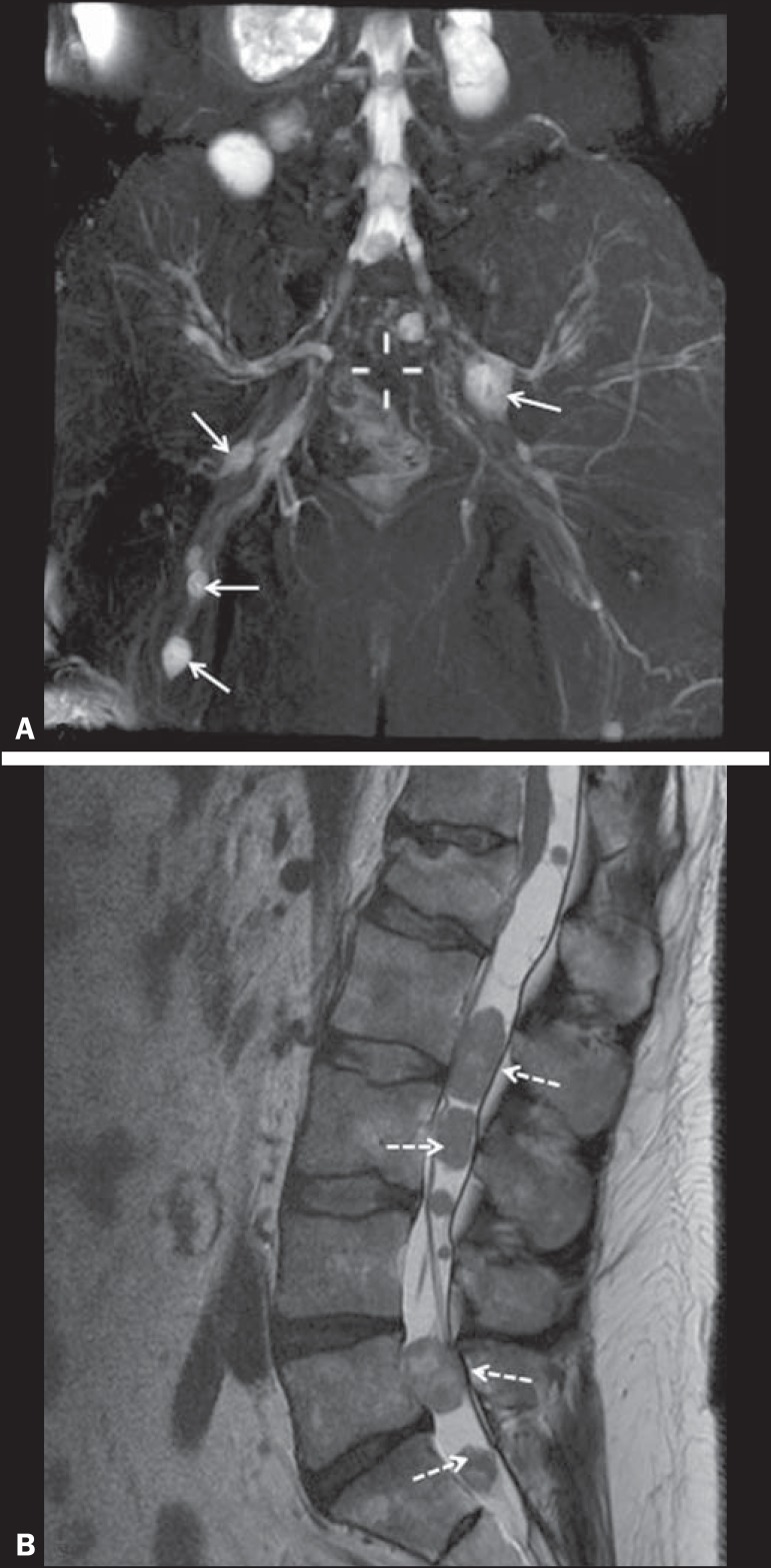
48-year-old male patient with schwannomatosis. **A:** Volumetric maximum intensity projection reconstruction of a coronal T2-weighted slice with fat saturation showing schwannomas in both sciatic nerves (arrows). **B:** Sagittal T2-weighted slice showing root schwannomas (arrows) within the spinal canal.

**Figure 3:**
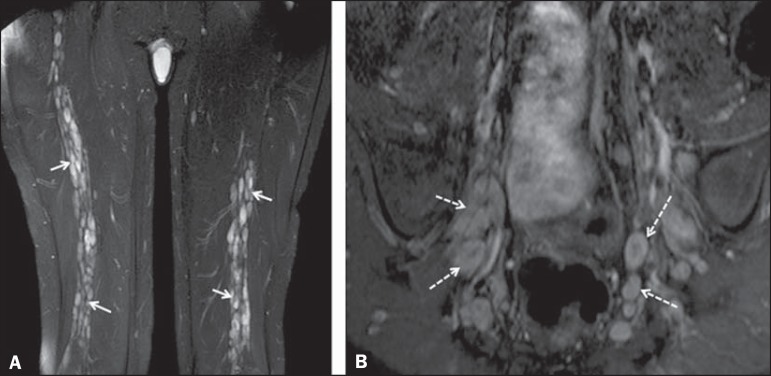
30-year-old male patient with neurofibromatosis type 1. **A:** Coronal T2-weighted slice with fat saturation, showing plexiform neurofibromas in both sciatic nerves (arrows). **B:** Volumetric maximum intensity projection coronal T2-weighted slice with fat saturation, showing neurofibromas at the roots of the lumbosacral plexus (arrows).

**Figure 4:**
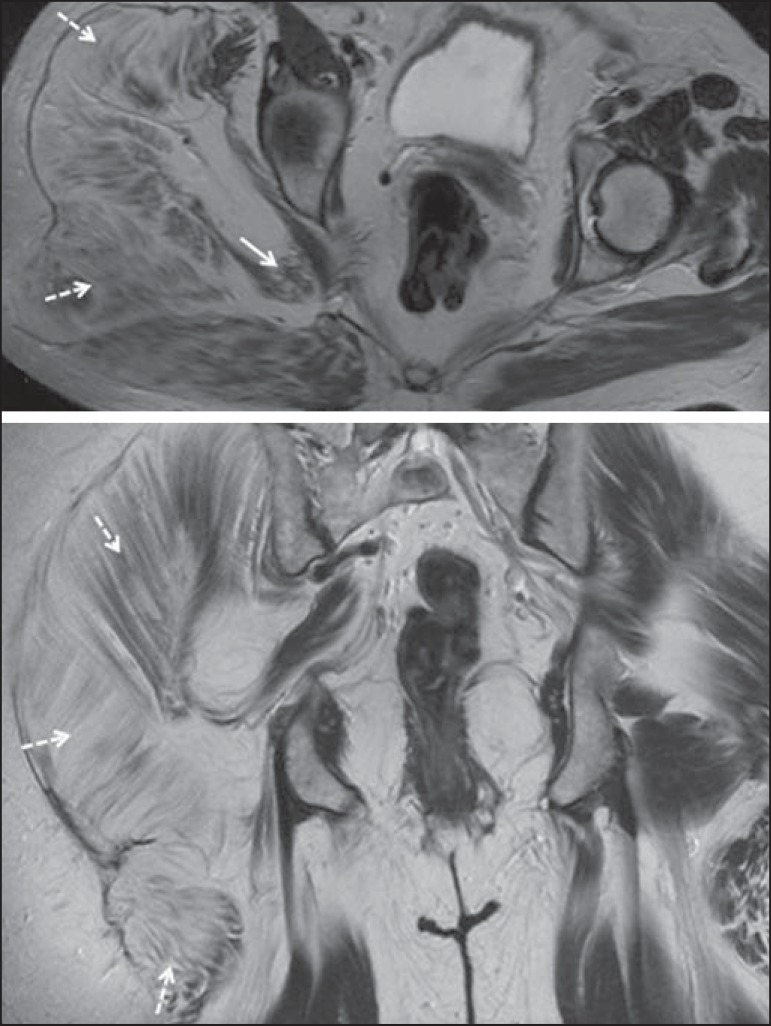
68-year-old female patient with increased volume of the right gluteal region and pain in the ipsilateral leg. **A:** Axial T2-weighted slice showing lipomatosis of the somatic nerve (solid arrow) accompanied by adipose infiltration into the ipsilateral pelvic girdle muscles (dashed arrows). **B:** Coronal T2-weighted slice of the pelvis, showing increased volume and adipose infiltration into the pelvic girdle muscles (arrows).

**Figure 5:**
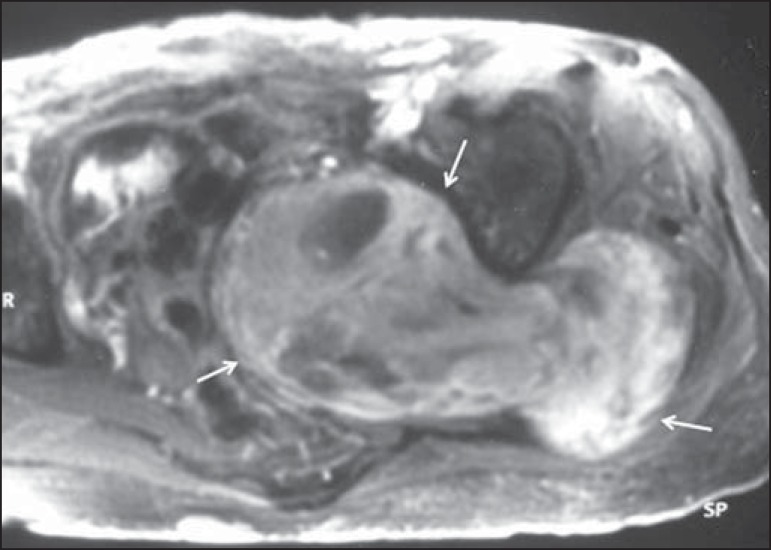
32-year-old male patient with a history of neurofibromatosis, presenting with pain in the left leg and enlargement of the left gluteal region. Contrast-enhanced axial T1-weighted slice showing an expansile dumbbell-shaped formation (arrows) on the sciatic nerve in the region of the sciatic notch, consistent with malignant peripheral nerve sheath tumor, as was confirmed by histopathology.

**Figure 6:**
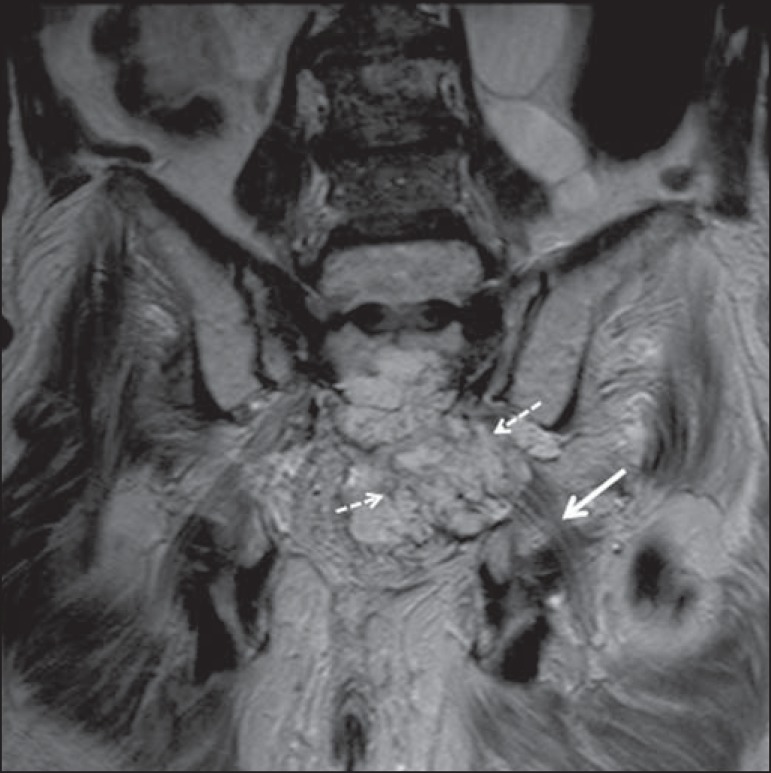
66-year-old male patient with rectal adenocarcinoma. Coronal T2-weighted slice showing an expansile lesion in the pelvic region, consistent with rectal adenocarcinoma, extending to the left sciatic notch (dashed arrows) and involving the right ipsilateral sciatic nerve (solid arrow).

**Figure 7:**
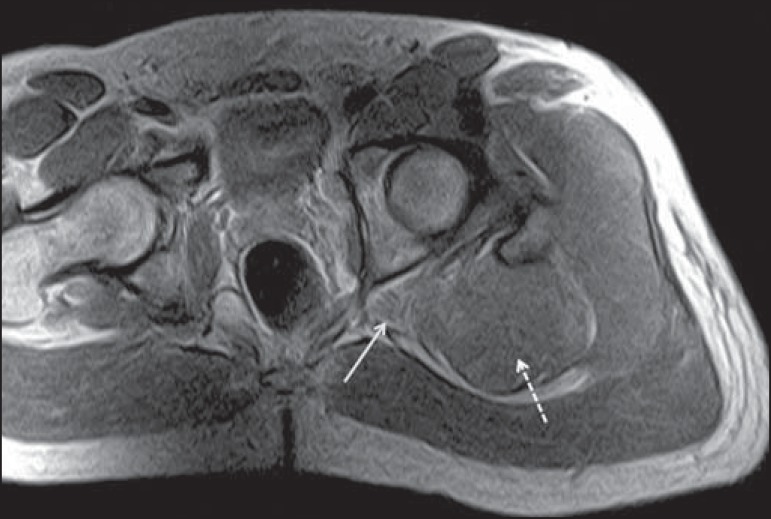
35-year-old male patient with a palpable mass in the left thigh. Axial T1-weighted slice showing a liposarcoma (dashed arrow) in the posterior compartment of the left thigh, compressing and displacing the ipsilateral sciatic nerve (solid arrow).

Benign or malignant neoplasms can arise from nerve fibers or from the cuff of the sciatic nerve sheath (Figures 2, 3, 4, and 5). However, the nerve can be affected because it is contiguous to or compressed by neoplasms in adjacent tissues (Figure 6 and 7). Another form of sciatic nerve involvement is perineural dissemination of neoplasms, which is particularly common in cases of prostate cancer^(11-13)^.

On MRN, a typical finding is an expansile lesion involving the sciatic nerve. Here, we illustrate examples of primary sciatic nerve neoplasms as well as secondary involvement of the sciatic nerve in cases of neoplasms in the region of the pelvis and proximal thigh.

## HEREDITARY CAUSES

Chief among the hereditary causes of sciatic neuropathies is Charcot-Marie-Tooth disease, which is a spectrum of diseases related to alterations currently described in more than 30 genes^(14)^. In imaging studies, the finding typical of Charcot-Marie-Tooth disease is diffuse nerve hypertrophy. Clinically, patients with Charcot-Marie-Tooth disease present muscular weakness, pain, and a variety of deformities related to muscular atrophy^(15)^.

**Figure 8:**
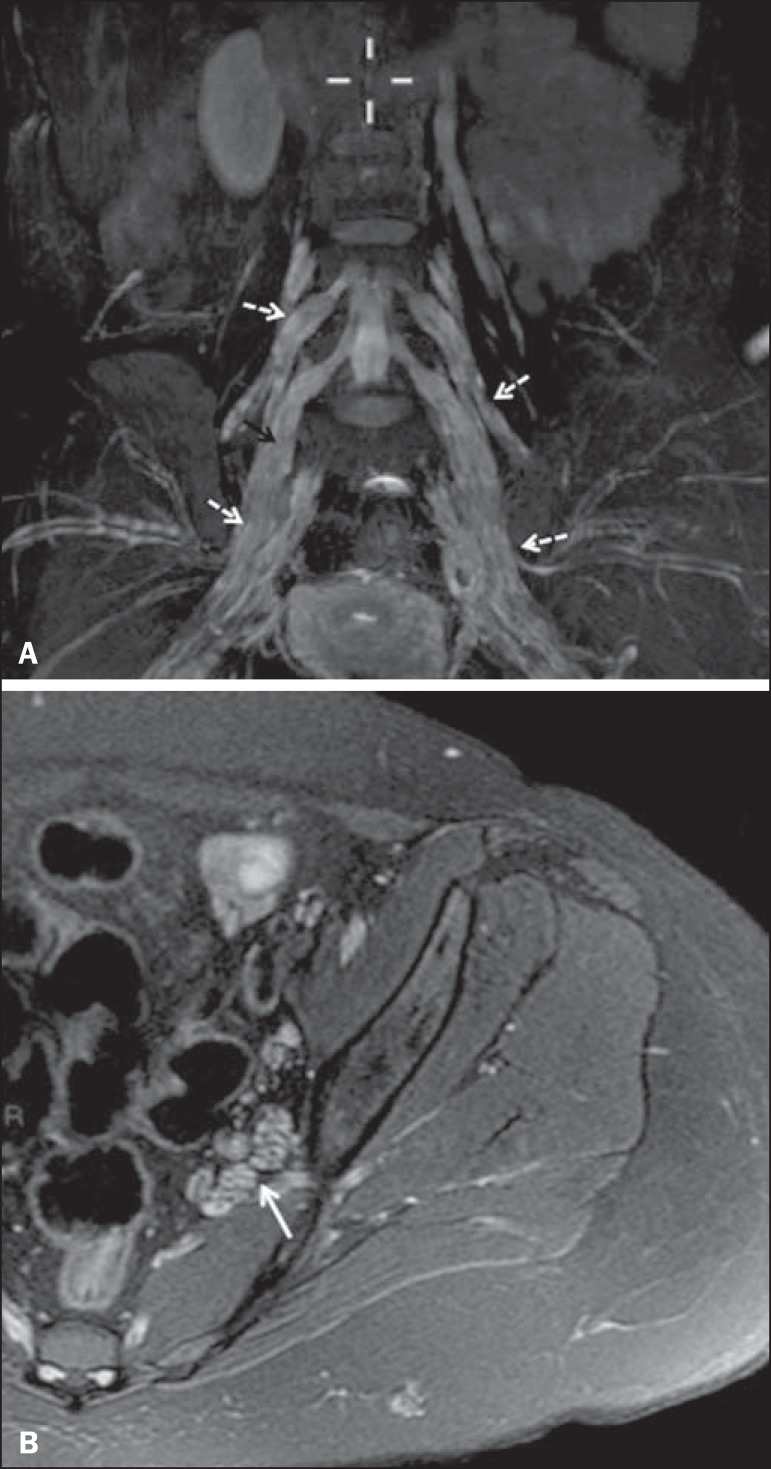
**A:** Volumetric maximum intensity projection reconstruction of a coronal T2-weighted slice with fat saturation, showing diffuse thickening of the roots of the lumbosacral plexus (arrows). **B:** Axial T2-weighted slice with fat saturation, showing a similar aspect in the left sciatic nerve (arrow).

Figure 8 illustrates the case of a 46-year-old patient with pain in the lower limbs and image findings typical of Charcot-Marie-Tooth disease.

## COMPRESSIVE CAUSES

As previously noted, the proximal segment of the sciatic nerve has an intimate relationship with the piriformis muscle. Variations include the sciatic nerve coursing anterior to, posterior to, or within the ventral portion of the piriformis muscle. When it is related to an abnormality in the piriformis muscle, neuropathy of the proximal segment of the sciatic nerve can be called piriformis syndrome, although there is controversy in the literature about the existence of this causal relationship^(16,17)^.

**Figure 9:**
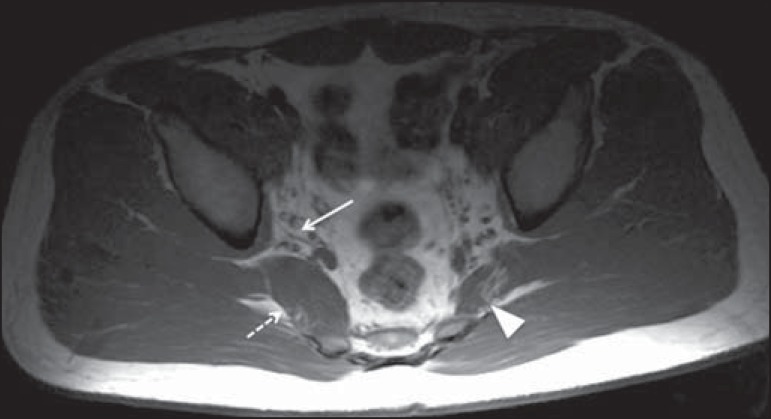
21-year-old male patient with pain in the right gluteal region and posterior right thigh. Axial T1-weighted slice showing hypertrophy of the right piriformis muscle (dashed arrow) and increased thickness of the ipsilateral sciatic nerve (solid arrow). Normal contralateral piriformis muscle (arrowhead).

**Figure 10:**
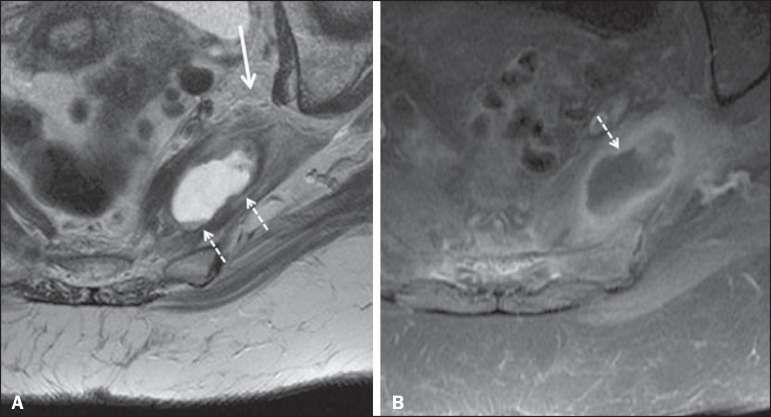
90-year-old patient with lower back pain radiating to the gluteal region and posterior surface of the left thigh. Axial T2-weighted slice (**A**) and contrastenhanced axial T1-weighted slice (**B**), showing an abscess in the left piriformis muscle (dashed arrows), resulting in reactive thickening of the ipsilateral sciatic nerve (solid arrow).

In compressive sciatic neuropathy, MRN findings include changes in the path, thickness, or signal of the sciatic nerve, with or without abnormalities of the piriformis muscle anatomy. We illustrate two examples of piriformis muscle alteration with neuropathy of the proximal portion of the sciatic nerve (Figures 9 and 10).

## TRAUMATIC CAUSES

Because of its particular anatomy and long course, the sciatic nerve can be affected by direct trauma or pelvic ring fracture, which are common in automobile accidents. Dislocations of the coxofemoral joint, which occur in cases of high-energy trauma, are also common causes of sciatic neuropathy^(18)^. In such cases, imaging findings include, other than the changes typical of neuropathy, alterations to the surrounding soft tissues, such as muscle bruising. The clinical correlation, if available, reveals a history of trauma, thus confirming the diagnosis.

**Figure 11:**
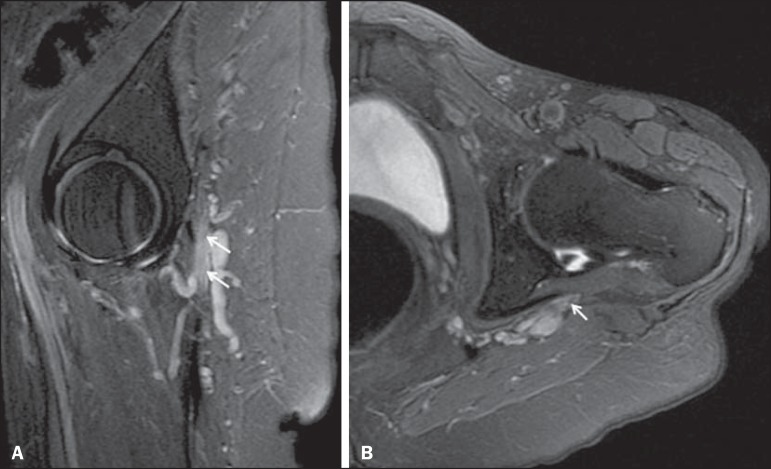
75-year-old female patient with pain in the left gluteal region after a fall from standing height. Sagittal T2-weighted slice (**A**) and axial T2-weighted slice (**B**), both with fat saturation, showing diffuse thickening of the left sciatic nerve with a hyperintense signal (arrows), consistent with traumatic neuropathy.

We illustrate a case of neuropathy caused by direct trauma resulting from a fall from standing height (Figure 11).

## IATROGENIC CAUSES

**Figure 12:**
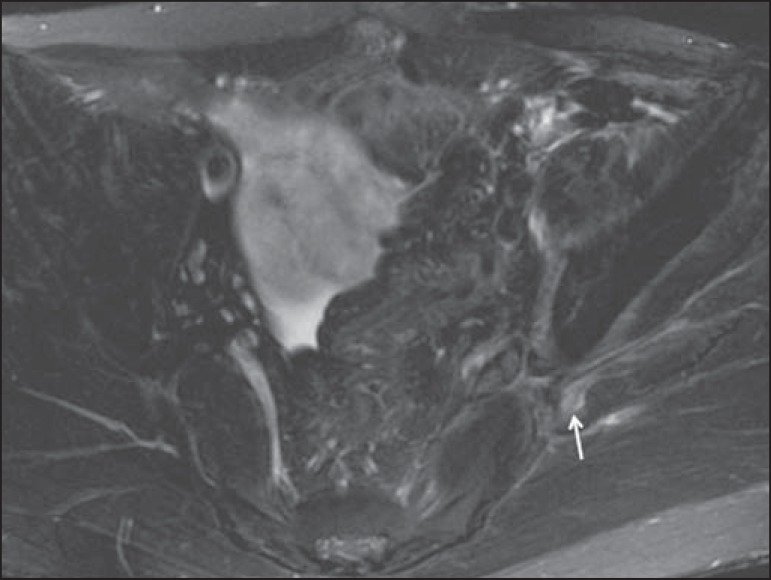
55-year-old male patient after pelvic radiotherapy for prostate cancer. Coronal T2-weighted slice with fat saturation, showing left sciatic nerve thickening with a hyperintense signal (arrow), consistent with radiation-induced neuritis.

One of the main iatrogenic causes of sciatic nerve neuropathy is radiotherapy for pelvic neoplasms, including prostate, gynecological, and colorectal cancer, resulting in radiation-induced neuropathy, as depicted in Figure 12^(11)^. The development of neural changes related to radiotherapy tends to occur at 5-30 months after treatment, its incidence peaking between months 10 and 20. In radiation-induced neuropathy, the imaging findings are nonspecific and include changes in the thickness and signal intensity of the affected nerve. The clinical correlation with a history of radiotherapy is fundamental to suggesting this diagnosis. Clinically, radiation-induced neuropathy is initially characterized by sensory manifestations, mainly pain and paresthesia, in some cases evolving to muscle weakness^(19-21)^.

**Figure 13:**
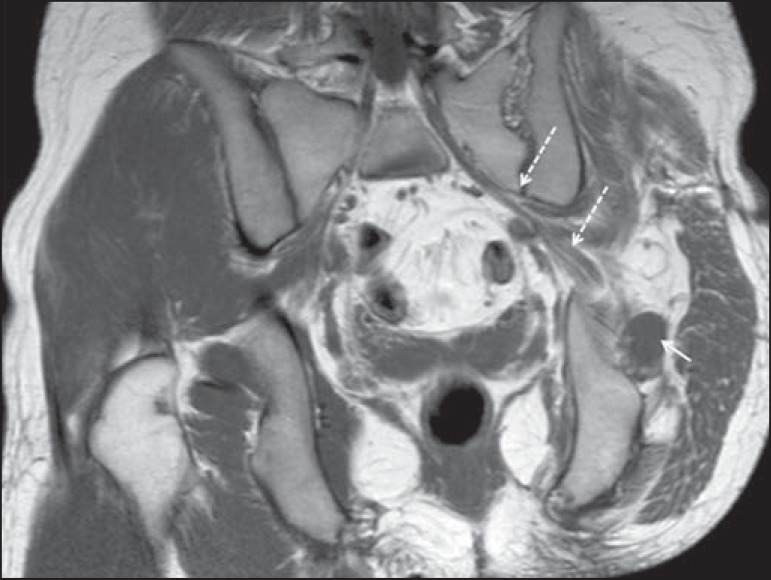
44-year-old male patient after surgical amputation of the left leg. Coronal T1-weighted slice showing a neuroma at the stump (solid arrow) of the left sciatic nerve (dashed arrows).

**Figure 14:**
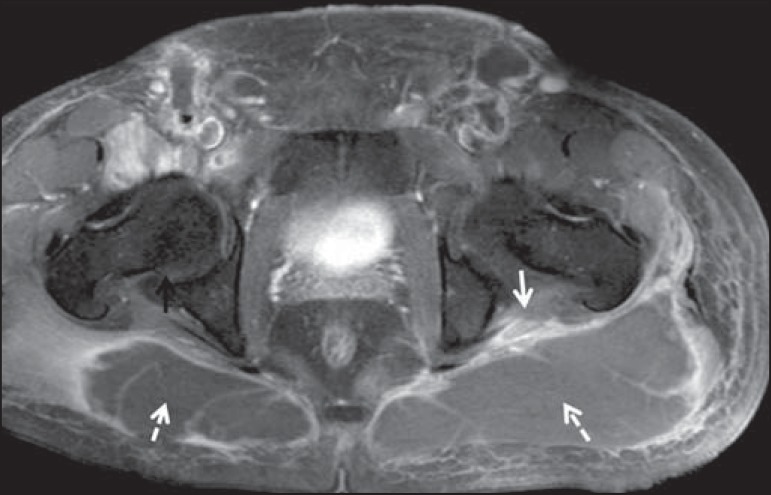
40-year-old male in the early postoperative period after placement of an aortoiliac stent. Axial T2-weighted slice with fat saturation, showing diffuse ischemia of the gluteal musculature (dashed arrows), together with thickening and signal alteration of the left sciatic nerve (solid arrow).

Neuroma (Figure 13) is a common sequela of amputation, causing pain that is usually refractory to pharmacological treatment and sometimes disabling. Although its pathophysiology is still poorly understood, there are measures that have proven effective in reducing its incidence. Such measures include implantation of the nerve stump into the ventral aspect of a muscle or capping the nerve stump with epineural flap. More rare and serious causes, such as ischemic neuropathy secondary to the placement of an aortoiliac stent^(22)^, can also be demonstrated (Figure 14). Like radiation-induced neuropathy, ischemic neuropathy presents nonspecific MRN findings, the clinical correlation also being fundamental.

## CONCLUSION

The sciatic nerve is often affected in diseases of the pelvis or lower limbs, as well as by lesions that originate within the nerve itself. Its course is long, which predisposes it to various types of compression. MRN is an important tool for the evaluation of peripheral nerve diseases and should be widely used for the study of the sciatic nerve whenever possible. Detailed knowledge of its anatomy and of the imaging aspects of the main diseases affecting it is fundamental to optimizing imaging studies of the sciatic nerve.

## References

[1] Petchprapa CN, Rosenberg ZS, Sconfienza LM (2010). MR imaging of entrapment neuropathies of the lower extremity.
Part 1. The pelvis and hip. Radiographics.

[2] Donovan A, Rosenberg ZS, Cavalcanti CF (2010). MR imaging of entrapment neuropathies of the lower extremity.
Part 2. The knee, leg, ankle, and foot. Radiographics.

[3] Chhabra A, Chalian M, Soldatos T (2012). 3-T high-resolution MR neurography of sciatic
neuropathy. AJR Am J Roentgenol.

[4] Soldatos T, Andreisek G, Thawait GK (2013). High-resolution 3-T MR neurography of the lumbosacral
plexus. Radiographics.

[5] Nucifora PG, Verma R, Lee SK (2007). Diffusion-tensor MR imaging and tractography: exploring brain
microstructure and connectivity. Radiology.

[6] Ono SE, Carvalho Neto A, Gasparetto EL (2014). X-linked adrenoleukodystrophy: correlation between Loes score and
diffusion tensor imaging parameters. Radiol Bras.

[7] Itagiba VGA, Borges R, Cruz Jr LCH (2010). Use of diffusion tensor magnetic resonance imaging in the
assessment of patterns of white matter involvement in patients with brain
tumors: is it useful in the differential diagnosis. Radiol Bras.

[8] Budzik JF, Balbi V, Verclytte S (2014). Diffusion tensor imaging in musculoskeletal
disorders. Radiographics.

[9] Li X, Chen J, Hong G (2013). In vivo DTI longitudinal measurements of acute sciatic nerve
traction injury and the association with pathological and functional
changes. Eur J Radiol.

[10] Sun C, Hou Z, Wan Q (2014). In vivo evaluation of sciatic nerve crush injury using diffusion
tensor imaging: correlation with nerve function and
histology. J Comput Assist Tomogr.

[11] Crush AB, Howe BM, Spinner RJ (2014). Malignant involvement of the peripheral nervous system in
patients with cancer: multimodality imaging and pathologic
correlation. Radiographics.

[12] Lim R, Jaramillo D, Poussaint TY (2005). Superficial neurofibroma: a lesion with unique MRI
characteristics in patients with neurofibromatosis type 1. AJR Am J Roentgenol.

[13] Lin J, Martel W (2001). Cross-sectional imaging of peripheral nerve sheath tumors:
characteristic signs on CT, MR imaging, and sonography. AJR Am J Roentgenol.

[14] Berciano J (2011). Peripheral neuropathies: molecular diagnosis of
Charcot-Marie-Tooth disease. Nat Rev Neurol.

[15] Morano JU, Russell WF (1986). Nerve root enlargement in Charcot-Marie-Tooth disease: CT
appearance. Radiology.

[16] Ozaki S, Hamabe T, Muro T (1999). Piriformis syndrome resulting from anomalous relationship between
the sciatic nerve and piriformis muscle. Orthopedics.

[17] Halpin RJ, Ganju A (2009). Piriformis syndrome: a real pain in the buttock?. Neurosurgery.

[18] Cornwall R, Radomisli TE (2000). Nerve injury in traumatic dislocation of the hip. Clin Orthop Relat Res.

[19] Todd M, Shah GV, Mukherji SK (2004). MR imaging of brachial plexus. Top Magn Reson Imaging.

[20] Fathers E, Thrush D, Huson SM (2002). Radiation-induced brachial plexopathy in women treated for
carcinoma of the breast. Clin Rehabil.

[21] Gikas PD, Hanna SA, Aston W (2008). Post-radiation sciatic neuropathy: a case report and review of
the literature. World J Surg Oncol.

[22] Lewin-Kovalik J, Marcol W, Kotulska K (2006). Prevention and management of painful neuroma. Neurol Med Chir (Tokyo).

